# Recent Shift in Climate Relationship Enables Prediction of the Timing of Bird Breeding

**DOI:** 10.1371/journal.pone.0155241

**Published:** 2016-05-16

**Authors:** Shelley A. Hinsley, Paul E. Bellamy, Ross A. Hill, Peter N. Ferns

**Affiliations:** 1 Centre for Ecology and Hydrology Monks Wood, Abbots Ripton, Huntingdon, Cambridgeshire, United Kingdom; 2 Cardiff School of Biosciences, BIOSI2, Cardiff University, Cardiff, United Kingdom; University of Vigo, SPAIN

## Abstract

Large-scale climate processes influence many aspects of ecology including breeding phenology, reproductive success and survival across a wide range of taxa. Some effects are direct, for example, in temperate-zone birds, ambient temperature is an important cue enabling breeding effort to coincide with maximum food availability, and earlier breeding in response to warmer springs has been documented in many species. In other cases, time-lags of up to several years in ecological responses have been reported, with effects mediated through biotic mechanisms such as growth rates or abundance of food supplies. Here we use 23 years of data for a temperate woodland bird species, the great tit (*Parus major*), breeding in deciduous woodland in eastern England to demonstrate a time-lagged linear relationship between the on-set of egg laying and the winter index of the North Atlantic Oscillation such that timing can be predicted from the winter index for the previous year. Thus the timing of bird breeding (and, by inference, the timing of spring events in general) can be predicted one year in advance. We also show that the relationship with the winter index appears to arise through an abiotic time-lag with local spring warmth in our study area. Examining this link between local conditions and larger-scale processes in the longer-term showed that, in the past, significant relationships with the immediately preceding winter index were more common than those with the time-lagged index, and especially so from the late 1930s to the early 1970s. However, from the mid 1970s onwards, the time-lagged relationship has become the most significant, suggesting a recent change in climate patterns. The strength of the current time-lagged relationship suggests that it might have relevance for other temperature-dependent ecological relationships.

## Introduction

The importance of large-scale climate processes in ecology is well established and especially so in recent decades in relation to global warming [1–5]. For western and northern Europe and eastern North America, the North Atlantic Oscillation (NAO) is one such climate process influencing both marine and terrestrial ecosystems [6,7]. In particular, the NAO winter index (NAO WI) (based on the difference in normalised sea pressures between the Azores and Iceland for December to March [8,9]) has been linked to breeding phenology, reproductive success and survival in a wide range of taxa including birds, mammals and fish [6,10,11]. In northern Europe, positive values of the index are associated with warmer, wetter winters and negative values with colder, drier ones, while the reverse is the case in North America (i.e. negative values, warm and wet; positive values, cold and dry) [8].

In temperate-zone birds, ambient temperature is an important cue enabling breeding effort to coincide with maximum food availability [12–14], and earlier breeding in response to warmer springs has been documented in many species [15,16]. It is a reasonable assumption that warmer springs are likely to follow warmer winters and thus a link between the NAO WI and bird breeding phenology in the immediately following spring might be expected and has indeed been demonstrated in both Europe and North America. For example, a significant negative relationship between the NAO WI and timing of breeding has been reported for several British great tit (*Parus major*) populations, i.e. breeding started earlier following a warmer winter [17], and recent work on European starlings (*Sturnus vulgaris*) in British Columbia found an earlier start to egg laying following warmer mid-winter temperatures [14]. Other studies have reported time-lags of one or more years in relationships with the NAO WI [6,10].

In this paper, we use 23 years of data for great tits breeding in deciduous woodland in eastern England to examine the effect of the NAO WI on timing of breeding. Instead of an expected direct effect of the immediately preceding winter [17], we found a novel relationship between the birds’ timing of breeding (measured as first egg dates) and the NAO WI such that timing can be predicted one year in advance, i.e. timing in year i is positively related to the NAO WI in year i-1. We also show how this relationship appears to be generated by a one year time-lag in an abiotic link between the NAO WI and local spring warmth in our study area. This time-lagged link has developed since the mid 1970s suggesting a recent shift in the large-scale climate processes influencing the birds’ breeding phenology.

## Methods

### Study sites

Our study area is located in Cambridgeshire in eastern England (52° 22’N, 0° 13’W) [18,19]. Breeding parameters were recorded for pairs of great tits and blue tits (*Cyanistes caeruleus*) using nest boxes in three study woods: Monks Wood (MW) National Nature Reserve 157 ha: 1993–2000, 22 boxes; 2001–2015, 35 boxes; Brampton Wood (BW), Wildlife Trust Nature Reserve, 132 ha: 1993–2015, 22 boxes; Wennington Wood (WW), privately owned, 72 ha: 1993–1999, 21 boxes; 2000–2015, 35 boxes). Most of the boxes are accessible (controlled by hole diameter: 32 mm both species, 25 mm blue tits only) to both species except in WW where 19 boxes are accessible to both and 16 (including all 14 erected after 2000) only to blue tits. Boxes are located in approximately one half of each of MW and BW and throughout WW giving densities of about 0.45, 0.33 and 0.49 boxes per ha respectively. This results in smaller numbers of study nests per wood than in some other long-term studies of tits [20], but we adopted this policy to reduce the risk of unduly increasing tit population densities which can affect timing of breeding [21] whilst also obtaining adequate sample sizes. However, from the mid 1990s BW has also contained c. 200 dormouse boxes which, until 2013 when they were redesigned, could be accessed by blue tits which probably increased the blue tit population in this wood. The main tree species are common ash (*Fraxinus excelsior*), English oak (*Quercus robur*) and field maple (*Acer campestre*) with an understory chiefly of hawthorn (*Crategus* spp.), hazel (*Corylus avellana*) and blackthorn (*Prunus spinosa*). BW also has some blocks of conifers but the boxes are located in deciduous areas.

### Timing of breeding

Timing of breeding was recorded for 23 years from 1993 to 2015 and was defined as the laying date (expressed as April 1^st^ = 1) of the first egg of the clutch. Tits typically lay one egg per day, early in the morning, and begin incubation on the day the last egg is laid to achieve synchronous hatching (although in poor weather delayed incubation after clutch completion is common, 22). The average clutch size in woodland is 9 for great tits and 11 for blue tits [23]. We inspected boxes approximately weekly during the laying period and calculated first egg dates by counting back from the number of eggs observed [24]. We included only nests which were active at the time of observation (i.e. at least one more egg was laid) and only those of first breeding attempts. For each wood, we calculated an annual mean first egg date (NERC-Environmental Information Data Centre http://doi.org/10.5285/2efa9bf4-e5c0-42f9-8fcb-90dca2bb9c66) [25]. For great tits, the mean (± SD) and range of the numbers of nests included in the calculation of the annual mean first egg date for each wood were: MW, 18 ± 5.0, range = 12–28; BW, 15 ± 2.8, range = 11–20; WW, 10 ± 2.1, range = 6–14. The numbers of blue tits using the boxes were smaller (MW, 8 ± 3.3, range = 3–13; BW, 3 ± 1.3, range = 2–6; WW, 9 ± 3.0, range = 5–15) because, as the larger of the two species at c. 19 g versus 10 g for blue tits, great tits are usually dominant. The results for blue tits were similar to those of great tits, but due to the small sample sizes per year we include here only those for great tits. All bird data were collected with the permission of the landowners (MW: Natural England; BW: The Wildlife Trust; WW: the Abbots Ripton Estate) by licenced (British Trust for Ornithology Ringing Scheme, permit nos. A4101 and C3288) persons experienced in the running and maintenance of nest boxes.

### Climate data and statistical analysis

Values for the NAO WI for the 23-year study period from 1993 to 2015 were obtained from the Climate Analysis Section, National Centre for Atmospheric Research (NCAR), Boulder, USA, [9] https://climatedataguide.ucar.edu/climate-data/hurrell-north-atlantic-oscillation-nao-index-station-based. Relationships between the annual mean timing of breeding of great tits in each wood and the NAO WI were then examined for the study period using least squares linear regression (Minitab Release 15). We used the NAO WI for the immediately preceding winter and also for one and two year time-lags, i.e. year i, year i-1 and year i-2. To test the predictive power of the one year time-lagged relationship for each wood, we used a jackknife approach to derive a mean % error (± SE) for the prediction of mean first egg date, i.e. we omitted each year sequentially and used the remaining 22 years to predict the timing of the missing year.

Timing of breeding in tits is known to be earlier in warmer springs [26,27], thus we also examined the relationships between the birds’ timing and local spring warmth in our study area, and between local spring warmth and the NAO WI (for year i and year i-1) using least squares regression. For our measure of local spring warmth, we used the ‘warmth sum’ calculated as the sum of maximum daily temperature for 1^st^ March to 25^th^ April [28]. This index was found to be the most useful of several indices tested by Perrins and McCleery [29] in relation to great tit breeding parameters. Although the time period of 1^st^ March to 25^th^ April is fixed whilst the average onset of breeding can vary by as much as 3–4 weeks between years depending on weather conditions, using a fixed index provides an unbiased measure of local spring warmth in relation to the NAO WI. We also examined how the warmth sum and/or the timing of breeding were related to the annual change, calculated as current year index minus previous year index, in the NAO WI. We used this annual change as an attempt to link events back to the previous year because, although it was clear how the immediately preceding NAO WI might have an influence, it was not clear how such an influence might act across time in the event of a one-year time-lag (but see discussion). We also investigated if winter or early spring rainfall might influence timing of breeding. We used a general linear modelling approach with site as a random factor, and warmth sum, NAO WI, rainfall and year as covariates. Rainfall was entered separately as the total monthly rainfall for the months of December, January, February and March, and was also modelled as combined winter rainfall, i.e. the total for December to March combined. We could not use year as a factor because, to avoid pseudoreplication, our first egg dates are average values for each wood for each year. However, year can be used as a covariate and this is also appropriate in the context of global warming promoting earlier breeding [15,16]. Maximum daily temperatures, and total monthly rainfall, for the 23-year study period were obtained from a UK Met Office weather station located approximately 50 m from the south side of Monks Wood (and approximately 4 km from WW and 9 km from BW).

To investigate the link between the warmth sum and the NAO WI in the longer term, we used a 23-year sliding window, with annual stepping, and data going back to 1878 [8,30,31] to generate 116 linear regressions of warmth sum on NAO WI (for year i and year i-1). Values for the NAO WI and maximum daily temperatures from 1878 were obtained respectively from the Climate Analysis Section, National Centre for Atmospheric Research (NCAR), Boulder, USA, [7] and the Met Office Hadley Centre Central England Temperature (HadCET) dataset, where daily maxima are available from 1878 [31]. The warmth sum calculated from the Monks Wood meteorological station data is a little warmer than that calculated using the HadCET data (for the period 1993 to 2015, mean, ± SD: Monks Wood, 686.6 ± 79.3; HadCET, 644.2 ± 78.9; mean difference, 42.2 ± 15.4; paired t-test, t = 13.34, *P* < 0.001), but the two data sets are highly correlated: r = 0.981, *P* < 0.001. In addition to the regression analyses, all other statistical comparisons were also carried out in Minitab Release 15.

## Results

### NAO WI and timing of breeding

**Fig 1:**
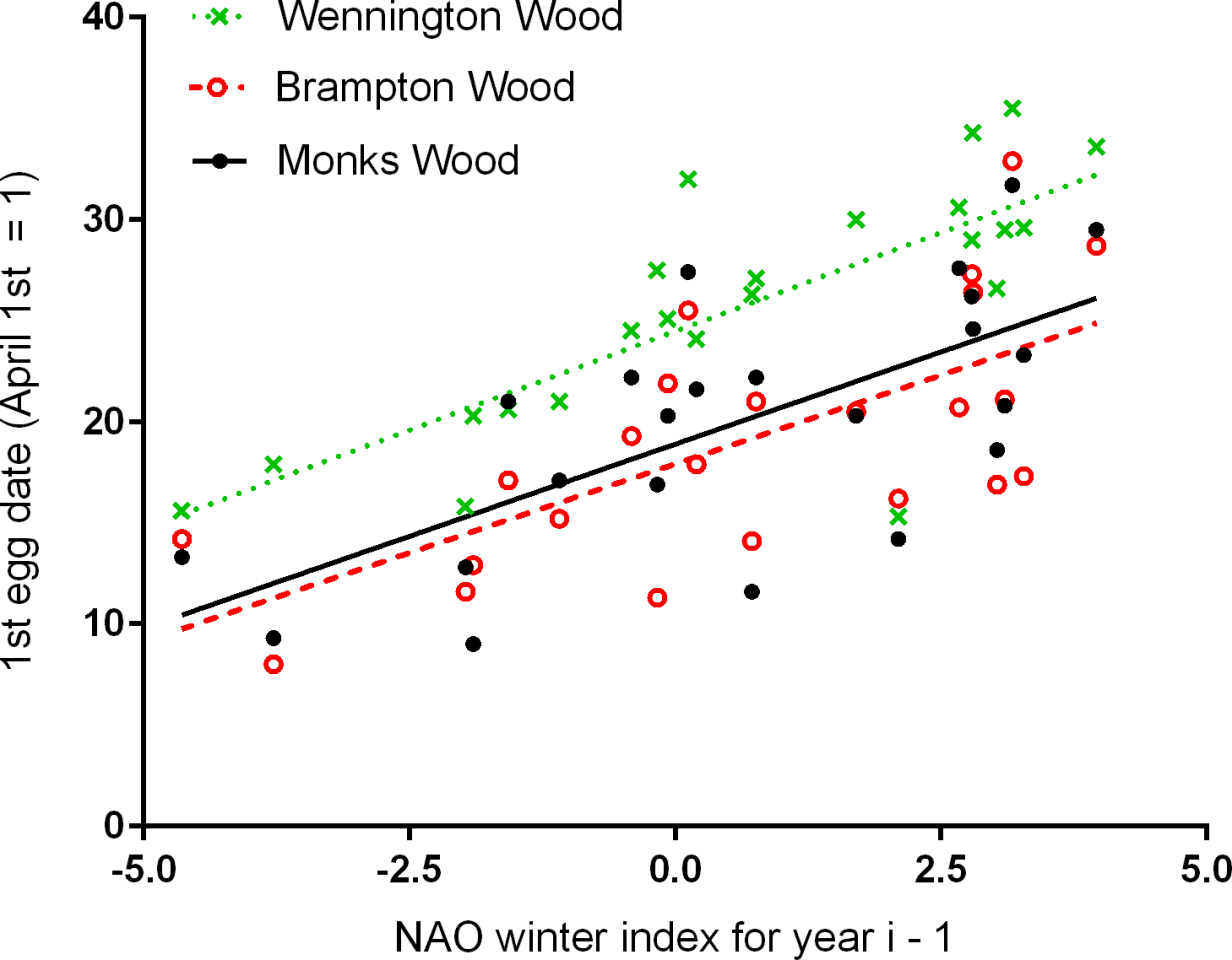
Time-lagged relationships between the timing of breeding (1^st^ egg dates) and the NAO winter index. Values of the NAO WI are for the winter of the year before, e.g. NAO WI for 2013/14, mean annual egg dates for 2015. Least squares regression equations are: MW 1^st^ egg date = 18.9 + 1.82 lagged NAO WI, R^2^ = 0.468, *P* < 0.001; BW 1^st^ egg date = 17.9 + 1.76 lagged NAO WI, R^2^ = 0.463, *P* < 0.001; WW 1^st^ egg date = 24.5 + 1.95 lagged NAO WI, R^2^ = 0.563, *P* < 0.001.

**Fig 2:**
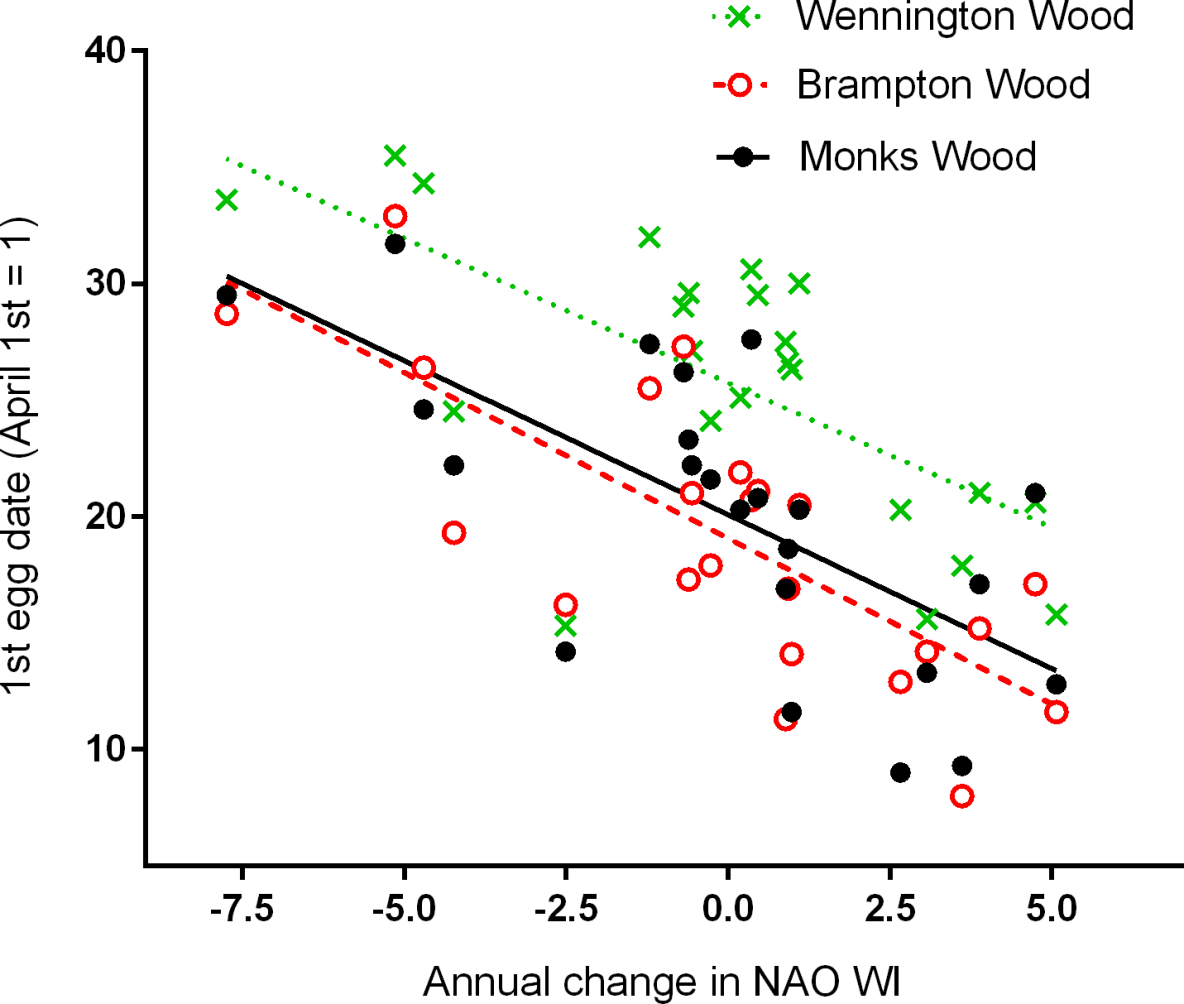
Relationships between the timing of breeding (1^st^ egg dates) and the annual change in the NAO winter index. The change in the NAO WI is calculated as the current year minus the previous year. Least squares regression equations are: MW 1^st^ egg date = 20.1–1.32 NAO WI change, R^2^ = 0.462, *P* < 0.001; BW 1^st^ egg date = 19.1–1.42 NAO WI change, R^2^ = 0.568, *P* < 0.001; WW 1^st^ egg date = 25.7–1.24 NAO WI change, R^2^ = 0.441, *P* = 0.001.

Timing of breeding in all three woods was related to both the one year time-lagged NAO WI (Fig 1) and the annual change in the NAO WI (Fig 2). Overall, the strengths of the relationships with timing using either the one year time-lagged NAO WI or the annual change were similar, but using the NAO WI for the preceding year, we can putatively predict timing of breeding one year in advance (e.g. NAO WI for 2013/14 predicts first egg dates for spring 2015). Using jackknife cross-validation, the mean % errors (± SE) of predicted first egg dates were: Monks Wood: 6.92 ± 1.44; Brampton Wood: 8.51 ± 1.77; Wennington Wood: 3.02 ± 0.63. Overall, the relationships with the lagged NAO WI tended to over-estimate the mean dates, i.e. predicted a later start to breeding than that observed. There were no significant relationships using the NAO WI for the immediately preceding winter (for MW, BW, WW respectively, R^2^ = 0.059, 0.122, 0.021; *P* = 0.263, 0.103, 0.513, all negative slopes), nor for a two year time-lag (R^2^ = 0.042, 0.025, 0.032; *P* = 0.347, 0.468, 0.411, all negative slopes).

### Local spring warmth, rainfall and timing of breeding

**Fig 3:**
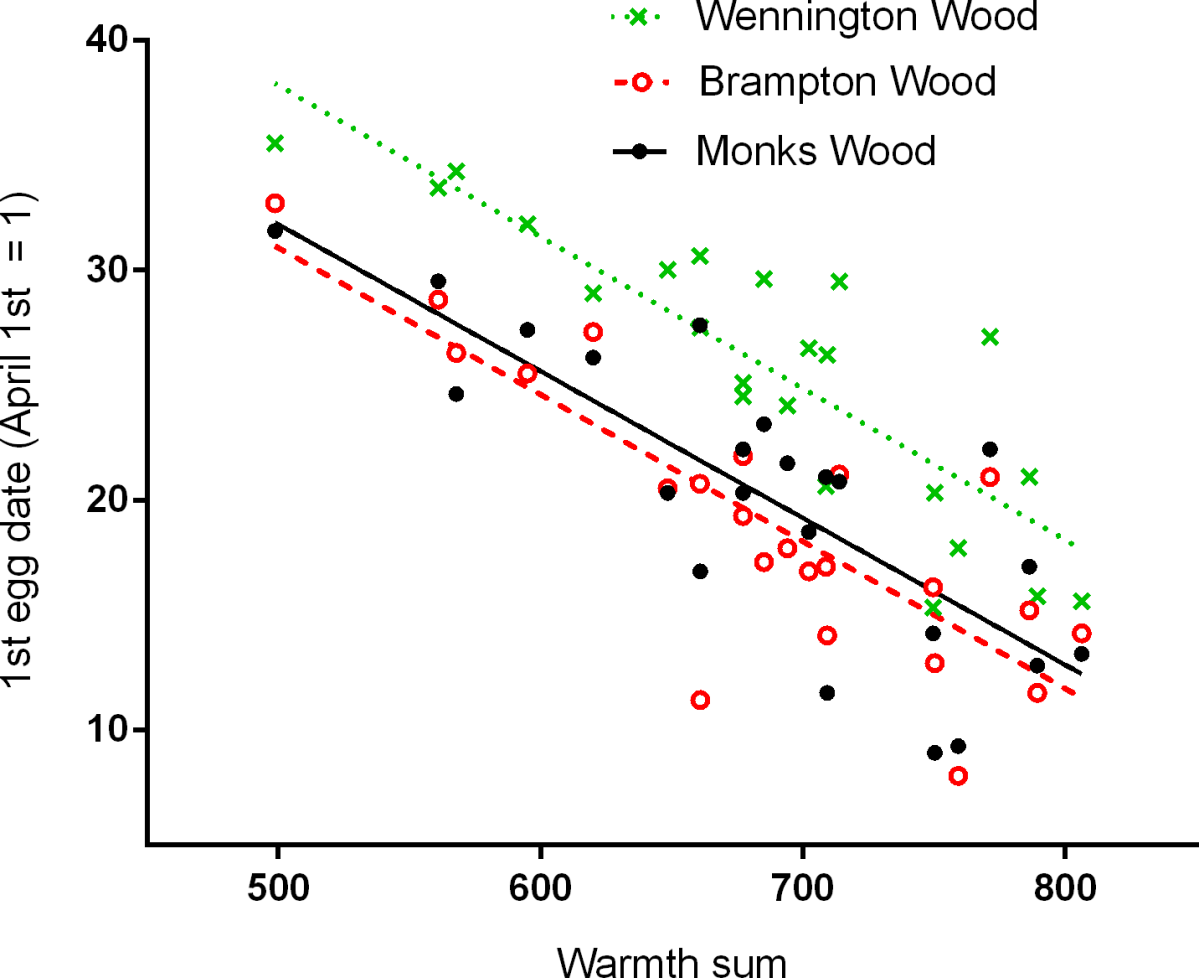
Relationships between timing of breeding (1^st^ egg dates) and local spring warmth (warmth sum). Larger values of the warmth sum reflect warmer springs and earlier laying. Least squares regression equations are: MW 1^st^ egg date = 63.9–0.0639 warmth sum, R^2^ = 0.640, *P* < 0.001; BW 1^st^ egg date = 62.9–0.0639 warmth sum, R^2^ = 0.681, *P* < 0.001; WW 1^st^ egg date = 71.0–0.0659 warmth sum, R^2^ = 0.739, *P* < 0.001.

Timing of breeding by great tits in all three study woods was linearly related to local weather conditions, measured by the warmth sum, such that egg laying started earlier in warmer springs (Fig 3). The relationships for all three woods were similar in slope, and for MW and BW also in intercept, but the latter differed for WW where, on average, breeding started consistently later (c. 6–7 days) than in the other two woods (ANOVA, F_(2,66)_ = 7.81, *P* = 0.001). In the models examining the influence of rainfall on timing of breeding, rainfall in the preceding months of December, January, February and March had no significant effect either separately or when combined as total winter rainfall. The month of December came closest with a non-significant delaying effect (F_(1,62)_ = 3.06, *P* = 0.085). The best overall model included year because of a significant interaction with site, Wennington Wood having significantly later egg dates than the other two woods (see above), but the effect of year on its own was not significant (F_(1,61)_ = 3.50, *P* = 0.066). Despite the strong effect of warmth sum on egg dates (F_(1,61)_ = 63.31, *P* < 0.001), the time-lagged NAO WI had an additional effect (F_(1,61)_ = 16.41, *P* < 0.001) and the total variance explained by the model was 82.3%.

### NAO WI and local spring warmth

**Fig 4:**
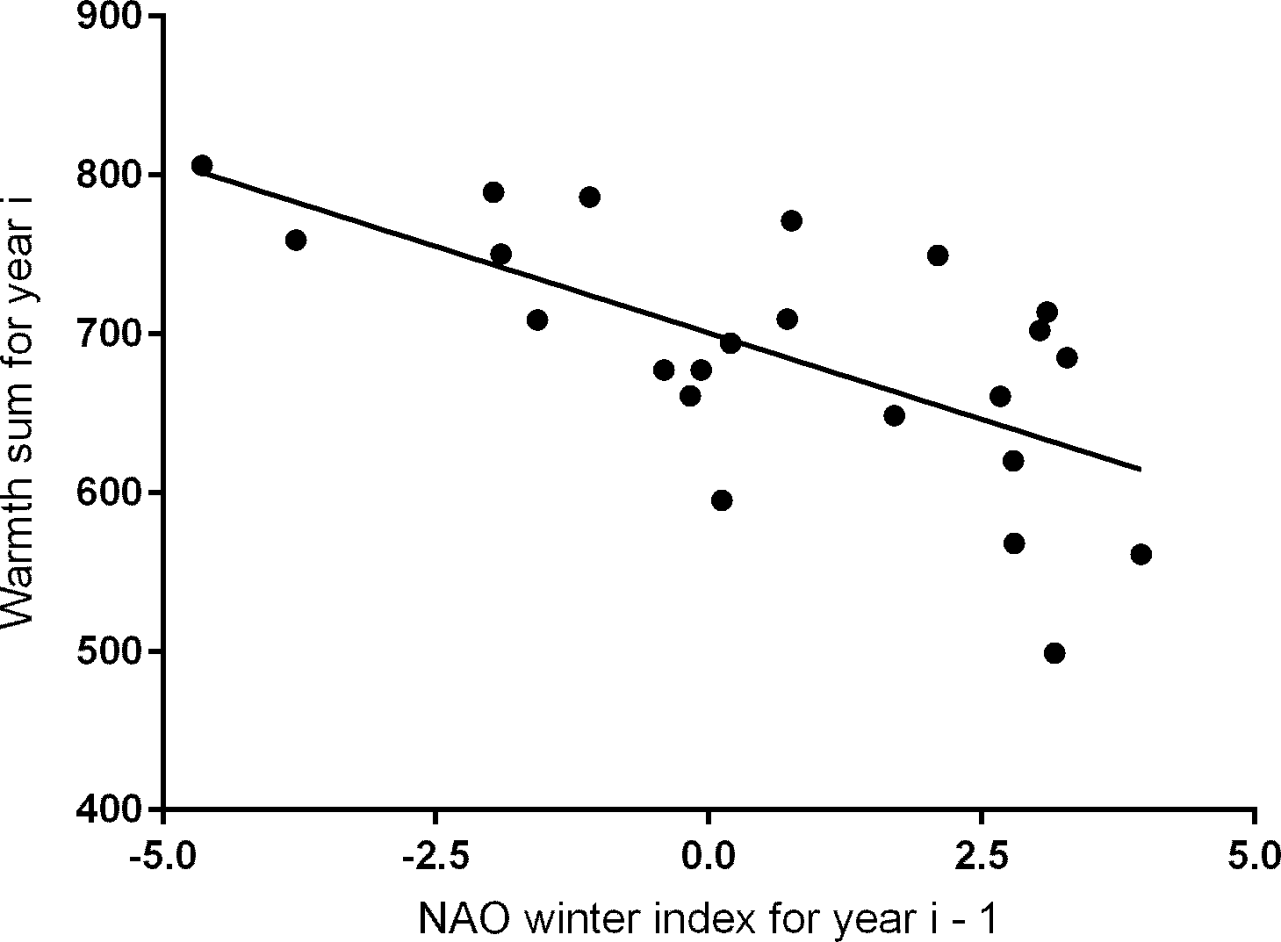
Time-lagged relationship between local spring warmth (warmth sum) and the NAO winter index. Values of the NAO WI are for the winter of the year before, i.e. warmth sum for year i, NAO WI for year i-1. Least squares regression equation is: warmth sum_i_ = 701–21.8 NAO WI_i-1_, R^2^ = 0.426, *P* < 0.001.

**Fig 5:**
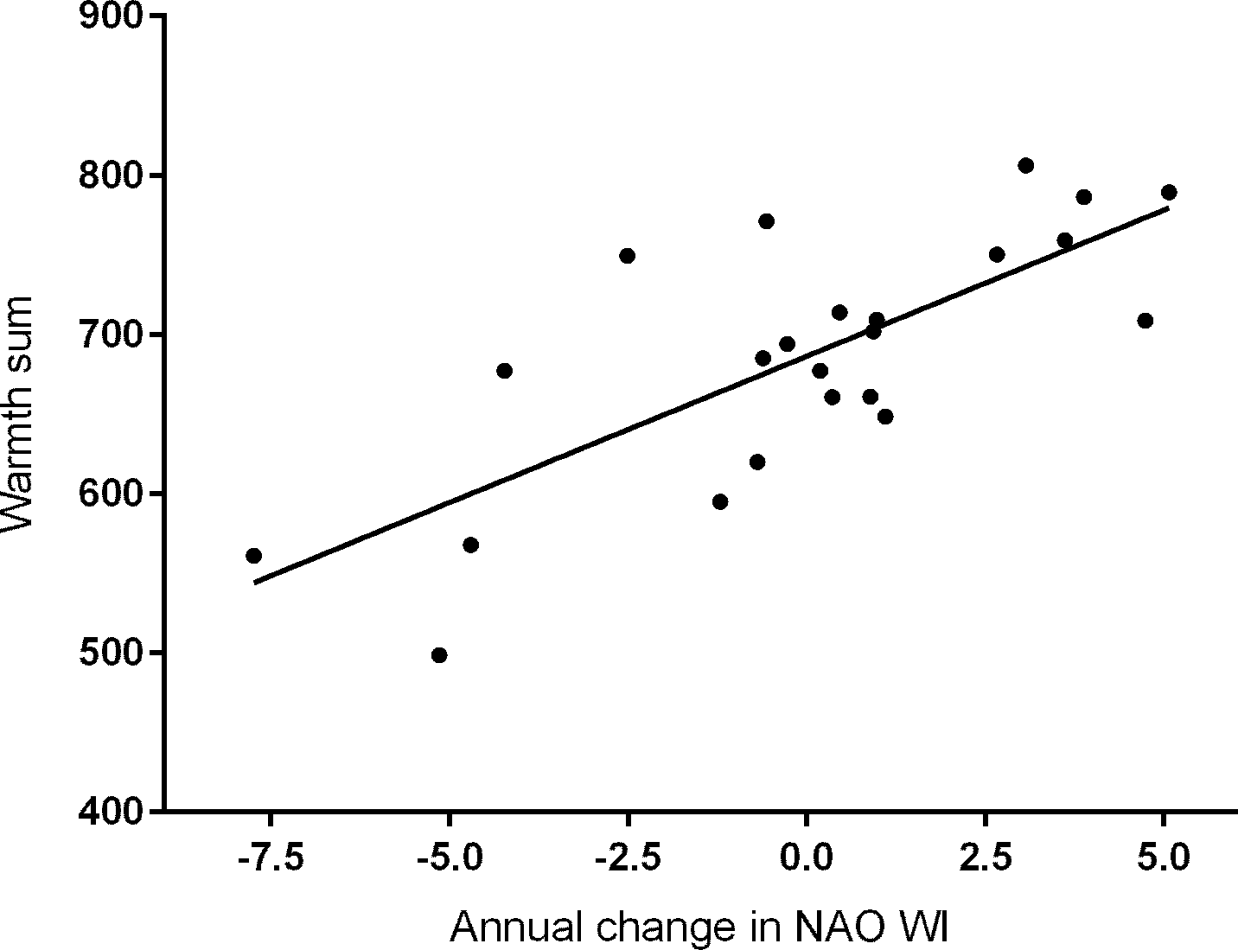
Relationship between local spring warmth (warmth sum) and the annual change in the NAO winter index. The change in the NAO WI is calculated as the current year minus the previous year. Least squares regression equation is: warmth sum = 686 + 18.4 NAO WI change, R^2^ = 0.568, *P* < 0.001.

For our 23-year study period, local spring weather, measured as the warmth sum, showed a significant (R^2^ = 0.426, *P* = 0.001) negative relationship with a one year time-lagged NAO WI, i.e. warmth sum for year = i and NAO WI for year = i-1 (Fig 4). In contrast, there was a positive relationship between the warmth sum and the NAO WI for the immediately preceding winter, i.e. warmer springs were associated with positive values of the NAO WI, but the relationship was not significant (warmth sum = 678 + 12.5 NAO WI, R^2^ = 0.141, *P* = 0.077). There was also a positive relationship between the warmth sum and the annual change in the NAO WI (Fig 5), but this relationship was significant (R^2^ = 0.568, *P* < 0.001) with a greater R^2^ value than the relationship with the time-lagged NAO WI. Thus greater fluctuation in the NAO WI, i.e. larger positive changes between years, were associated with warmer springs, which is consistent with current observations of both warmer and more extreme weather in the UK [32].

**Fig 6:**
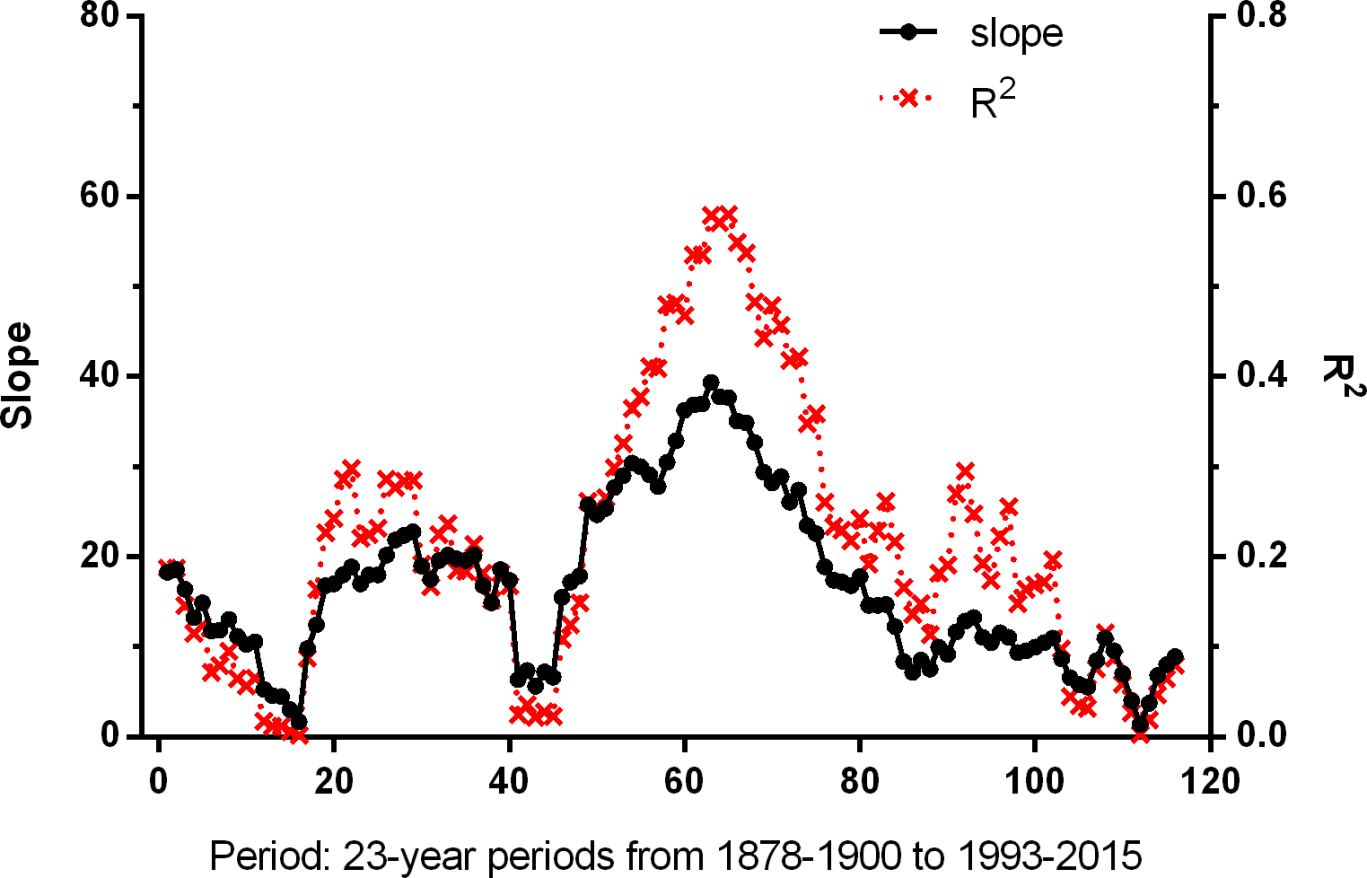
Parameters (slope and R^2^) of the linear regression relationships between the warmth sum and the current year, i, NAO WI (i.e. no time-lag) for 116 successive 23-year periods. Period 1 = 1878–1900; Period 116 = 1993–2015 (current study period). The relationships were significant (*P* ≤ 0.05) in Periods 1 and 2, Periods 19 to 37 and 39, and Periods 49 to 84, 89 to 97, 101 and 102. The slopes of the relationships were all positive.

**Fig 7:**
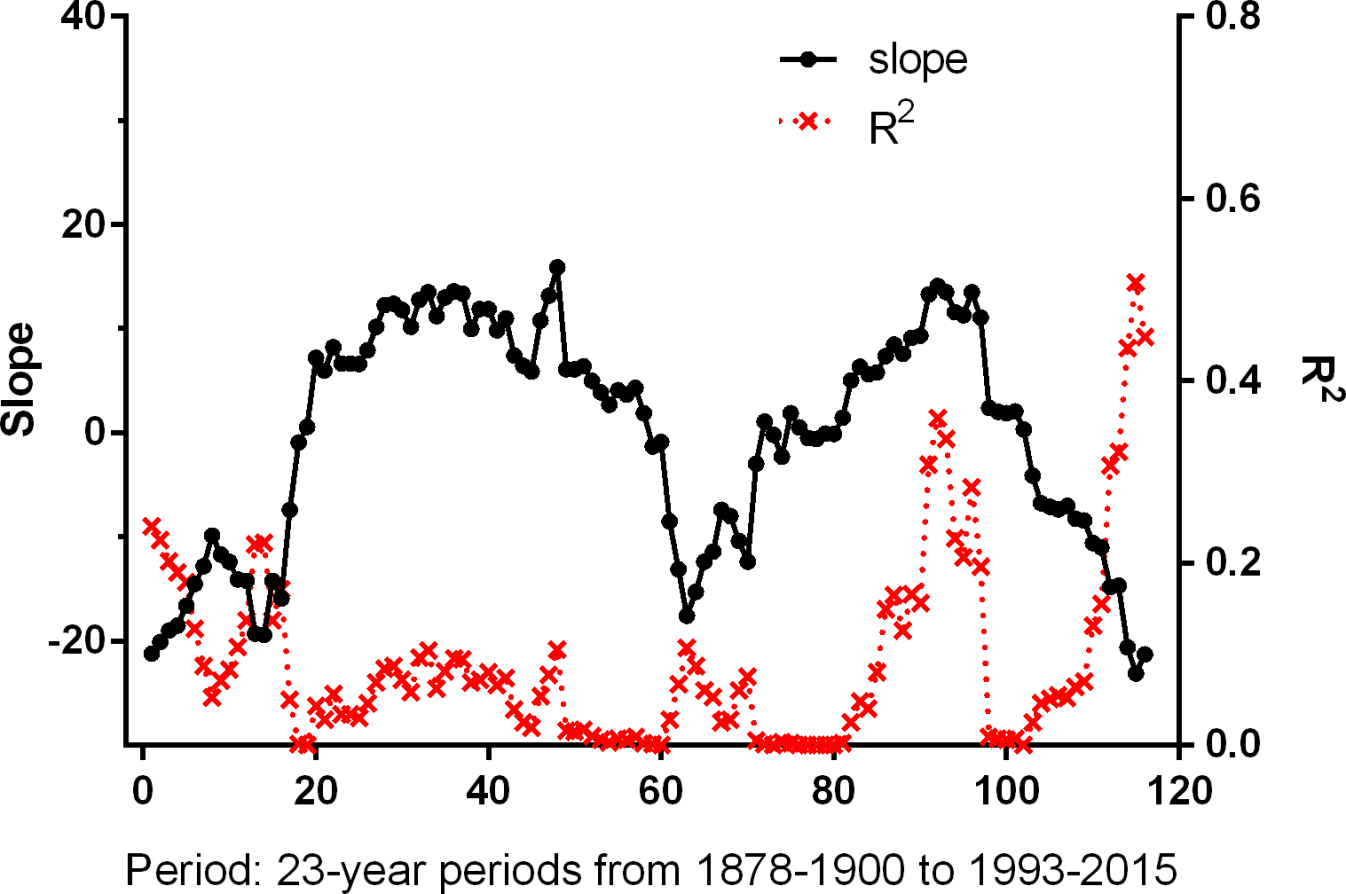
Parameters (slope and R^2^) of the linear regression relationships between the warmth sum and the previous year, i-1, NAO WI (i.e. with time-lag) for 116 successive 23-year periods. Period 1 = 1878–1900; Period 116 = 1993–2015 (current study period). The relationships were significant (*P* ≤ 0.05) in Periods 1 to 5, Periods 13, 14 and 16, Periods 91 to 97 and Periods 112 to 116. Slopes were both positive and negative, with negatives slopes becoming more frequent in the latter half of the dataset (since c. 1950).

Using a 23-year sliding window to examine the relationships between the warmth sum and the NAO WI in the longer term showed that, in the past, significant relationships with the immediately preceding NAO WI (Fig 6) were more common than those with the time-lagged NAO WI (Fig 7), and especially so from the late 1930s to the early 1970s (Periods 49 to 84). This span of years includes the years (1948 to 1957) when Sanz [17] found significant relationships between the immediately preceding NAO WI and timing of breeding in some great tit populations. However, more recently, from the mid 1970s (about Period 87) onwards, the time-lagged relationship has become the most significant which is suggestive of a change in climate patterns in recent years (although given the smoothing effect of this approach, these results must be interpreted carefully). There was no significant correlation between the NAO WI in successive years, either in the whole of the period 1878–2015, or in the 23-year periods in which there were highly significant relationships between the NAO WI and the warmth sum (Ljung-Box tests, Q ≤ 2.6, *P* > 0.10 in all cases).

## Discussion

It is well known that many bird species, including great tits, begin breeding activity earlier in warmer springs [15,16,33]. However, to the best of our knowledge, this is the first time that a relationship with climate has been shown to enable prediction of the likely timing of breeding one year in advance. Although our results are for the date of egg laying in great tits, the prediction can be applied to spring phenology in general (in eastern England at least) because earlier breeding activity in warmer springs is related to temperature-dependent development of vegetation and invertebrate food supplies. Great tits attempt to exploit the spring peak in defoliating arboreal caterpillars to rear their young [13,34–36] and the timing, abundance and duration of maximum caterpillar availability depends on factors, such as tree bud burst and leafing phenology, which are influenced by temperature and other environmental conditions [22,37–40]. Rainfall did not significantly influence timing, probably because wet springs are also usually cold [22] and, overall, temperature has by far the greatest influence.

The onset of breeding in tits can typically vary by up to about a month between years; across all three of our woods, the mean annual first egg date varied by a maximum of 28 days across the 23 years. Despite this variation, our predictive ability was generally robust, R^2^ values for the relationships between timing and the lagged NAO WI in the three woods ranged from 0.463 to 0.563 with P < 0.001 in all cases (Fig 1). Using these relationships and the NAO WI value for 2014/15 (i.e. 3.56), average (± SD) first egg dates for 2016 for MW, BW and WW respectively are predicted as 25^th^April (± 7.6 days), 24^th^ April (± 7.4) and 1^st^ May (± 6.5), which, as in 2015, will be another relatively late spring, five days later than the overall 23-year mean dates. However, despite the general observation that breeding in birds starts earlier in warmer years, the response to temperature, and other conditions, is likely to be more complex than this ‘rule of thumb’ implies [21,38–42] and may account for the unexpectedly early start to breeding in WW in 2009 (c. two weeks earlier than predicted). In contrast to this early start, the usually consistent later start to breeding in WW was probably indicative of habitat differences, for example, timing is known to become later as wood size declines [24] and WW has a higher proportion of ash trees than either MW or BW which may have implications for the abundance and diversity of invertebrate food supplies [43].

Influences of large-scale climate processes in ecology are well established [1–7] and have been used to examine historical events as well as to predict future ones. For example, in an analysis of great tit population dynamics spanning 100 years in Switzerland [11], breeding phenology and performance were linked to a chain of events starting with global climate processes (measured using winter/late spring NAO indices and the North Sea–Caspian Pattern), proceeding through local weather patterns (rainfall and temperature) and habitat phenology (beech bud burst) and ending with individual life histories. A number of studies have also reported time-lags in ecological responses to the NAO, but these are usually driven by biotic rather than abiotic factors. For example, lags of several years in annual cohort sizes of cod (*Gadus morhua*) have been recorded with both negative and positive relationships in different populations depending on local sea conditions [6]. These effects were driven by biotic links between the NAO WI, local sea temperatures and high recruitment following maturation of particular generations following increased survival of younger, immature stages in previous years. Similarly, abundance of red deer (*Cervus elaphus*) at Sør-Trøndelag in Norway and on the Isle of Rum in Scotland both showed a two year lag in relation to the NAO WI [10], although the biotic mechanisms underlying these relationships (increases in annual fecundity and\or changes in over-winter survival) differed between the two locations.

In a recent review of climate impacts on wildlife populations [44] it was concluded that indirect biotic mechanisms have greater impact than abiotic ones (the work concentrated on long-term effects, with a minimum data run of 20 years, and did not consider short-term extreme events, [22]). Such complexity is well demonstrated by golden plover (*Pluvialis apricaria*) population sizes in the English Peak District where a decline in numbers showed a two year time-lag in relation to the NAO [45] and local weather conditions. Numbers of tipulids, the main prey item for plover chicks, could be reduced by as much as 95% due to larval mortality in dry weather in the previous year, causing low recruitment and hence plover population decline in the following year [46]. Other studies [17, 47, 48] have shown a direct link between the immediately preceding NAO WI and timing of breeding which is consistent with breeding starting earlier under warmer conditions. For the 23 years of our study, despite a strong direct relationship between first egg dates and local spring warmth (Fig 3), there was no significant (*P* = 0.077) direct link with the NAO WI for the immediately preceding winter, although the relationship showed a distinct positive trend, i.e. warmer springs were associated with positive values of the NAO WI. Given the strong relationship between the timing of breeding and the warmth sum, and between the warmth sum and both the annual change in the NAO WI (Fig 5), and its value in the previous year (Fig 4), it seems likely that there is a time-lag in abiotic factors linking the large-scale climate processes to the local spring weather rather than a biotic link.

The observation that positive values of the NAO WI are associated with warmer and wetter winters in northern Europe provides a plausible link to the warmth sum in the immediately following spring, but how the NAO WI might influence the warmth sum with a one year delay is unclear. The sliding window analysis suggested that conditions causing the delay are a relatively recent development. Factors influencing large-scale climate processes are complex, for example, the range of factors influencing the El Niño Southern Oscillation (ENSO) include solar radiation, melting of ice sheets, natural fluctuations in greenhouse gases, human induced global warming and various feedback mechanisms [49,50]. Thus it may not be surprising that full development of an El Niño can be delayed for a year, e.g. the event predicted for 2014 eventually developed in 2015 and is now set to last until mid 2016 [51]. Concerning our study, the Atlantic Multidecadal Oscillation (AMO) is an index based on sea surface temperatures in the Atlantic Ocean [52]. Sea surface temperatures influence air temperature in the UK, and the AMO has been found to correlate well with the occurrence of dry and wet periods in North America [53]. Furthermore, recent work suggests that the NAO may be influenced by the AMO across a 10 to 15 year timescale with potential for decadal forecasting [54]. Thus interactions between ocean, atmospheric and land temperatures may involve substantial time-lags, but how this might influence our findings is currently only speculation, and relationships with the AMO merit further investigation. Whatever the cause of the current lagged relationship between spring warmth in eastern England and the NAO WI, it appears to be indicative of recent change in larger-scale processes underlying and associated with the NAO WI consistent with general observations of changing global climate. Other recent work has also documented such change, for example, Schmidt et al. [4] describe a climate change-related shift in a long-term relationship between seabird population dynamics and ocean conditions, including a greater influence of large-scale El Niño events on survival than local ocean conditions. Thompson and Ollason [55] also highlight the potential for changes in the effects of climate systems on long-lived seabirds to take years to become apparent. The current strength of our time-lagged relationship between the warmth sum and the NAO WI suggests that it might have relevance for other temperature-dependent ecological relationships [56].
